# Antiproliferative and Antimicrobial Activities of Secondary Metabolites and Phylogenetic Study of Endophytic *Trichoderma* Species From *Vinca* Plants

**DOI:** 10.3389/fmicb.2018.01484

**Published:** 2018-07-11

**Authors:** Sahar Leylaie, Doustmorad Zafari

**Affiliations:** Department of Plant protection, Faculty of Agriculture, Bu-Ali Sina University, Hamedan, Iran

**Keywords:** endophytic *Trichoderma* species, trichodermin, volatile compounds, antimicrobial, anti-proliferative

## Abstract

Endophytic fungi have been recognized as a potential source of bioactive secondary metabolites. The endophytic *Trichoderma* species were isolated from *Vinca* plants (*Vinca major, Vinca herbacea*, and *Vinca minor*), found in Iran and screened for antimicrobial and anti-proliferative activity. Based on morphological and phylogenetic analyses, four fungal species were identified: *T. asperellum, T. brevicompactum, T. koningiopsis, and T. longibrachiatum*. In addition, endophytic fungi bioactivity of methanol and ethyl acetate extracts (7.8–250 μgml^−1^) were assessed against a panel of pathogenic fungi and bacteria and IC_80_ was calculated. Data showed that both methanol and ethyl acetate extracts from all endophytic isolates had significant cytotoxic effects against the model target fungus *Pyricularia oryzae*. Further research indicated that they had significant antimicrobial bioactivity against the human pathogenic bacteria *Staphylococcus aureus* and *Escherichia coli*, and plant pathogenic bacteria *Ralstonia solanacearum* and *Clavibacter michiganensis* as well. According to the bioactivity results, crude ethyl acetate extract of *T. koningiopsis* VM115 isolate was determined for TLC and GC-MS analysis. An antifungal compound was isolated from ethyl acetate extract of *T. koningiopsis* VM115 based on bioassay guided fractionation. The ^1^H-NMR and ^13^C-NMR spectroscopic data showed that the compound was trichodermin, which exhibited strong fungicidal effects against *P. oryzae, Aspergillus fumigatus*, and *Botrytis cinera* with MICs of 31.25 μg ml^−1^ through *in vitro* antifungal tests. GC-MS analysis identified six classes of volatile compound produced by *T. koningiopsis* VM115 (alcohols, esters, pyrones (lactones), acids, furanes and lipids). 6-n-pentyl-6H-pyran-2-one (6PP) was identified as one of the most abundant metabolites in this research. These results indicate that the fungal endophytes from *Vinca* plants had antibacterial and cytotoxic activities; evidence that endophytes are a good source of biological activity and compounds. This work is the first report of Trichodermin production by *T. koningiopsis* species.

## Introduction

Although a large number of secondary metabolites are produced by endophytic fungi, which are non-essential, this can serve as an ecological advantage to them in certain environments. Pharmaceutically useful compounds, pigments, plant growth regulators, and mycotoxins are included in the mentioned metabolites (Keller et al., 2005). Thus, identification of these components and optimization of fungal growth conditions can help to achieve maximum production of secondary metabolites. The fungal genus *Trichoderma* contains some of the most potent biocontrol agents in use today (Harman et al., 2004; Ming et al., 2012). Moreover, some of its taxa have been shown to occur as endophytes, especially in tropical arboreous vegetation, the strains of which have often high antagonistic activities against their pathogens (Fravel, 1988). *Trichoderma* species are generally regarded as saprophytic fungi; as such they have minimal nutritional needs and produce and secrete a plethora of secondary metabolites into their vicinity. Though these compounds often appear in an organism with obscure or unknown functions, they can have medical, agricultural, and industrial applications, thus being important for humans. *Trichoderma* spp. displays antimicrobial activity against many important bacteria, yeasts, and filamentous fungi (Vizcaíno et al., 2005), in which numerous and varied secondary metabolites, such as peptaibols, gliotoxin, gliovirin, polyketides, pyrones, and terpenes may be involved (Schnürer et al., 1999; Vinale et al., 2008). Structural consideration of the *Trichoderma* antibiotic molecules present in nature has identified two main types (Sivasithamparam and Ghisalberti, 1998; Reino et al., 2008): volatile metabolites and low molecular weights like simple aromatic compounds, some polyketides including pyrones, isocyanates, butenolides, and volatile terpenes, all of which are rather non-polar substances of considerable vapor pressures; and polar metabolites of high molecular weights, which may induce direct interactions between *Trichoderma* spp. and their antagonists in the same way as gliovirin, peptaibols, and diketopiperazine-like gliotoxin. As a natural product, terpenes constitute the largest group of secondary metabolites with important pharmacological activities such as antiviral, antibacterial, antimalarial, and anti-inflammatory actions, inhibition of cholesterol synthesis, and anticancer activity. A large series of these compounds are produced by the filamentous fungi like *Trichoderma* (Degenkolb et al., 2008; Reino et al., 2008; Korpi et al., 2009; Cardoza et al., 2011; Mukherjee et al., 2012). Sesquiterpenes from *Trichoderma* have demonstrated antibacterial, antifungal and neuroleptic activities (Bennett and Klich, 2003). One particular group of sesquiterpenes includes fungal toxins known as trichothecenes. There are different species of *Trichoderma* producing bioactive compound that act as a mycotoxin such as Trichothecene. It is a sesquiterpenoid derived secondary metabolite synthesized mainly by *Fusarium*, other fungal genera such as *Trichoderma, Trichothecium, Stachybotrys* (Wilkins et al., 2003; Shentu et al., 2014; Frisvad and Thrane, 2004). It is known that these sesquiterpenoid compounds are also harmful to plants and animals that feed on infected fodder. Also, Trichothecenes in *Trichoderma*, trichodermin, and harzianum A, have been reported by only some species (*T. arundinaceum* and *T. brevicompactum*), (Corley et al., 1994; Cardoza et al., 2011). Trichodermin displays antifungal and anti-yeast activities as well as phytotoxicity (Nielsen, 2003; Rocha et al., 2005; Jin et al., 2007; Tijerino et al., 2011). Reino et al. (2008) have reviewed many volatile secondary metabolites that can be potentially produced by *Trichoderma* spp. Volatile secondary metabolites have demonstrated a key role in mycoparasitism of *Trichoderma* and its interaction with plants (Vinale et al., 2008). *Trichoderma* species are known to produce more than 40 different metabolites besides many important secondary metabolites like mycotoxins, which induce antimicrobial activities (Sivasithamparam and Ghisalberti, 1998). As a well-described volatile product, 6-pentyl-a-pyrone (6-PAP) represents antimicrobial and herbicidal activities through a secondary metabolism in *Trichoderma* (Galindo et al., 2004). These metabolites have been utilized in different biological processes, including the bio-control of microorganisms with their living environments. They can be produced to induce a competition between species by mediating resistance against predators, parasites, and diseases, and facilitating reproductive processes (Sivasithamparam and Ghisalberti, 1998).

About 7 species originate from *Vinca* (*Apocynaceae*) genus worldwide. It has been represented by *Vinca herbacea* Waldst and Kit as a native plant with two other cultivated species, *Vinca mino*r L. and *Vinca major* L., in Iran (Rechinger, 1974; Mozaffarian, 2006). In central and southern Europe, as well as southwest of Asia, *V. minor* L. (lesser periwinkle) has been a native plant, while being cultivated in the US and other countries as a ground cover. *V. major*, with common names such as big leaf periwinkle, large periwinkle, greater periwinkle, and blue periwinkle; it is a flowering plant species native to western Mediterranean. *V. herbacea*, which is commonly called herbaceous periwinkle, is a native plant capable of flowering in the east and southeast parts of Europe. It is distributed from Austria toward Greece at the south and Crimea at the east; it is also found in north western Asia, in the Caucasus and Alborz mountain ranges. These plants have been traditionally applied worldwide to treat various ailments throughout the ages. The plants bear active phyto constituents and exhibit varied pharmacological activities such as anti-cancer, anti-diabetic, anti-oxidant, anti-hypertensive, anti-microbial, and cytotoxic activities (Kral, 2012).

The goal of this study was to screen for antimicrobial and anti-proliferative activity in endophytic fungi of *Trichoderma* species isolated from the surface of sterilized leaves and stems of three *Vinca* plants found in Iran; *V. major, V. herbacea*, and *V. minor*. Tests were done for significant anti-proliferative and antibacterial capabilities of endophytic *Trichoderma* species from *Vinca* plants. The aim was to identify secondary metabolites produced by superior isolates, using thin layer chromatography (TLC), gas chromatography combined with mass spectrometry (GC-MS), MS, ^1^H-NMR (Nuclear Magnetic Resonance), ^13^C-NMR. Being identified as trichodermin by using spectroscopic data, antifungal compounds were isolated through bioassay-guided fractionation.

## Materials and methods

### General procedures based on experiments

Column Chromatographies (CCs) of silica gel G (200–300 mesh, Merck.) and Sephadex LH-20 (Merck.) and TLC of silica gel GF254 (10–40 lm, Merck.) were performed. Distillation of all the solvents was done before use. NMR spectra and ESI-MS were obtained using a spectrometer (Bruker AM-400) and Finnigan LCQ-Advantage (m/z), respectively. The chemical shifts d (ppm) rel. to Me4Si, and coupling constants J (Hz.) were then conducted. All the other chemicals under study were of analytical grades.

### Sampled locations

Samples were collected from five distinct provinces of Iran, representing the versatility of the country, i.e., Esfahan (South of Iran), Mazandaran (North of Iran), Hamedan (West of Iran), Tehran (Center of Iran). Plant temple (stem, leaf) were collected from three species *V. major, V. herbacea*, and *V. minor* as listed in Table 1.

**Table 1:** Endophytic *Trichoderma* species isolates from each *Vinca* host plant species, according to sampling sites, plant tissue and fungal isolate.

**Location (Iran)**	**Plant host**	**Plant segment**	**Identification**	**Isolate**
Esfahan (Esfahan)	*Vinca minor*	Stem	*T. asperellum*	VM 100
32°34′57.19“N, 51°29′0.45″E, 27342m				
Esfahan(Esfahan)	*Vinca minor*	Stem	*T. longibrachiatum*	VM 99
31°23′57.16“N, 51°34′17.74″E, 2034m				
Mazandaran(Sari)	*Vinca minor*	Stem	*T. brevicompactum*	VM102
36°10′10.69“N, 52°45′51.34″E, 63749m				
Mazandaran(Sari)	*Vinca herbacea*	Stem	*T. longibrachiatum*	VH104
36°18′36.25“N, 52°22′59.60″E, 10450m				
Tehran(Tehran)	*Vinca major*	Stem	*T. brevicompactum*	VM98
35°44′22.56“N, 51°10′31.94″E, 429m				
Hamedan(Hamedan)	*Vinca major*	Stem	*T. longibrachiatum*	VM111
34°46′7.90“N, 48°30′46.03″E, 1270m			*T. koningiopsis*	VM115

*T, Trichoderma*.

### Recovery and identification of endophytic fungi

Fresh tissue was collected from *Apocynaceae*, in each locality from June to October, 2014. From each plant specimen, three randomly selected pieces were surface sterilized and in total 700 plant pieces (~0.5 × 0.5 × 1 cm from inner layers) were incubated on potato dextrose agar (PDA) and water agar (WA) culture media, at 26–28°C, for 2–12 weeks. Hyphal tips were isolated, purified, and maintained at 4°C. The endophytic isolates were identified by investigating their colony morphology and the mechanism of spore production on PCA (Potato Carrot Agar), after 7 days at 22–25°C, under 16/8-h light intervals. Fungal specimen was stained and studied under microscope, according to the reference (Bissett, 1984, 1991a,b, 1992; Gams and Bissett, 1998).

### Identification based on molecular and morphological features

The cultures were grown on 2% Malt Extract Agar (MEA) and Potato Dextrose Agar (PDA) at 20°C under an ambient daylight condition or in a light/dark cycle of 12 h/12 h under fluorescent and near-UV light. To determine linear growth rates, fresh mycelial plugs were placed near the edges of PDA plates with a diameter of 9 cm and incubated at 20°C. The colony radius was measured at an interval of 24 h. Using the preparations performed in lactic acid, microscopic observations and measurements were conducted. Following maturing of the conidia within 4–7 days of incubation, observation of the structures and morphologies of macronematous conidiophores taken from the edges of conidiogenous fascicles or pustules was made. After 14 days, conidial morphology was recorded and measurements were done. Based on a comparison with the keys and descriptions presented in recent taxonomic literature, morphologies of the preliminary species were identified through observation (Bissett, 1984, 1991a,b,c, 1992; Gams and Bissett, 1998; Hoyos-Carvajal et al., 2009). Molecular identification was based on the fungal isolate growth in the test tubes containing PDB media at 28°C for 7 days. Using CinnaPure-DNA (Sinaclon, Iran), the genomic DNA was extracted after harvesting the mycelium. A region of nuclear rDNA, containing the internal transcribed spacer regions 1 and 2 and the 5.8S rDNA gene region and a fragment of *tef1* was amplified by polymerase chain reaction (PCR) using the primer pair ITS1 (5′-TCCGTAGGTGAACCTGCGG-3′) and ITS4 (5′-TCCTCCGCTTA TTGATATGC-3′) and the primer pair *tef1* fw (5′-GTGAGCGTGGTATCACCATCG-3′) and *tef1* rev (5′-GCCATCCTTGGAGACCAGC-3′) was done by Kraus et al. (2004). The PCR products were sent to the Macrogen sequencing service (Macrogen Inc. Seoul, Korea) for direct sequencing of double strands of DNA.

### Phylogenetic analyses

Sequences were checked with BioEdit v. 7.0.9.0 (Hall, 2006). The ITS and EF1-α sequences of outgroup (*Hypomyces subiculosus* TFC 97-166) and additional isolate was retrieved from GenBank. Sequences were aligned with MUSCLE (Edgar, 2004). Manual adjustments were done if necessary after checking the alignments. Using the simple indel-coding implemented by GapCoder, the phylogenetic analyses were performed based on the phylogenetic information contained in the indels (gaps) (Young and Healy, 2003). Phylogenetic analyses were performed with PAUP v. 4.0b10 (Swofford, 2003) for neighbor-joining (NJ) and maximum-parsimony (MP) analyses as described by Abdollahzadeh et al. (2010, 2014). Bootstrap analysis was done with 1,000 replicates. The general time-reversible model of evolution (Rodriguez et al., 1990), including estimation of invariable sites and assuming a discrete gamma distribution with six rate categories (GTR+I+Γ) was used. A partition homogeneity test (PHT) was used to determine the congruence between the ITS and EF1-α datasets (Farris et al., 1995; Huelsenbeck et al., 1996; Abdollahzadeh et al., 2014). New sequences were deposited in GenBank (Table 2).

**Table 2:** Isolates used in this study.

**Strain no**.	**Identification**	**GenBank Accession number**	
		**ITS**	**EF1-α**
VM 100	*T. asperellum*	KY412854	KY412863
VM 99	*T. longibrachiatum*	KY412857	KY412862
VM102	*T. brevicompactum*	KY412860	KY425693
VH104	*T. longibrachiatum*	KY412859	KY425694
VM98	*T. brevicompactum*	KY412856	KY412861
VM111	*T. longibrachiatum*	KY412858	KY425692
VM115	*T. koningiopsis*	KY412855	KY425691
CBS 816.68	*T. longibrachiatum*	EU401556.1	AY865640.1
CBS 112447	*T. brevicompactum*	EU330942.1	EU338300.1
CBS 433.97	*T. asperellum*	AY380912.1	AY376058.1
PPRC J9	*T. longibrachiatum*	EU401564.1	EU401613.1
DAOM 229982	*T. koningiopsis*	EU280141.1	EU280028.1
DAOM 233971	*T. koningiopsis*	EU280131.1	EU280021.1
TFC 97-166	*Hypomyces subiculosus*	FN859452.1	FN868770.1
BF06	*T. brevicompactum*	KU851839.1	KU851841.1
TaR3	*T. asperellum*	KT001078.1	KT722735.1
CBS 142.95	*T. atroviride*	AF456917.1	AY376051.1
CBS 836.91	*T. reesei*	X93951.1	GQ354354.1

### Metabolite extraction

To cultivate the fungi on PDB, the selected endophyte cultures were inoculated in 250 mL Erlenmeyer flasks containing 100 mL of the medium. Each flask was incubated at 28°C for 2 weeks with periodical shaking at 150 rpm. After the incubation period, the fungal fermentation broth was homogenized by addition of 10% methanol. Methanol and ethyl acetate were used as organic solvents to extract the metabolite via the solvent extraction procedure. An equal volume of solvent was added to the filtrate, mixed well for 10 min and kept for 5 min until the two clear immiscible layers were formed. By using a separating funnel, separation of the upper layer of the solvent, which contained the extracted compounds, was done. To obtain the crude metabolite, the compound yielded by evaporating the solvent was dried in a rotator evaporator under vacuum (Bhardwaj et al., 2015; Sharma et al., 2016). A brown gum was presented after evaporation of the combined ethyl acetate and methanol extracts. The crude extract was then dissolved in dimethyl sulphoxide at 1 mgmL^−1^of concentration and was stored at 4°C for 24 h before injecting in to GC-MS and the secondary metabolites were kept at −20°C until they were needed for bioassays.

### Anti-proliferative activity

Anti-proliferative and cytotoxic bioactivity of methanol and ethyl acetate extracts was determined against the conidial germination of *P. oryzae*, as a model. *P. oryzae* conidial suspension (4 × 104 mL^−1^; 50 μL including 0.02% yeast extract) was seeded into each well of the 96-well microtiter plates. To obtain the final concentrations of 250, 125, 62.5, 31.25, 15.62, and 7.81 μg mL^−1^, each well received 50 μL of the sample extract in a serially dilution manner. The assay plates were incubated at 28°C for 16 h. For each sample extract, a microscopic observation was done on the germinations and sizes of the germ tubes that originated from the 75 conidia and the results were compared with the control group to determine MICs. The experiments were performed in triplicate (Kobayashi et al., 1996).

### Cell viability assay

For the measurement of cell viability, tetrazolium salt MTT was applied to determine the fungal methanol and ethyl acetate extracts cytotoxicity (μg/mL^−1^) against *P. oryzae* conidia(Levitz and Diamond, 1985; Patel et al., 2013). Our methodology was based on a catalyzed reaction of the functional hyphae through hydrogenases, which led to the cleavage of the yellow tetrazolium salt MTT [3-(4,5-dimethylthiazol-2-yl)- 2,5-diphenyltetrazolium bromide] to MTT-formazan as its purple derivative. MTT-formazan can be quantified through spectrophotometry within 550 nm after being dissolved in isopropanol. A 50 μL of the conidial suspension of *P. oryzae* (7.5 × 104 mL^−1^) including a 0.02% yeast extract together with a 200-μl aliquot of conidial suspension was poured into each well of a 96-well plate to yield a final concentration of 15,000 conidia/well. An untreated conidial suspension of fungal extract extracted from *P. oryzae* was used as the control.

### Minimal inhibitory concentration (MIC) and minimum bactericidal concentration (MBC) determination

Methanol and ethyl acetate extracts prepared from the endophytes were examined for their antimicrobial activity against Human pathogenic bacteria gram-positive *Staphylococcus aureus* PTCC (1189) and Gram-negative *Escherichia coli* PTCC (1399) and Plant pathogenic bacteria Gram-negative *Ralstonia solanacearum* and Gram-positive *Clavibacter michiganensis* (laboratory of bacteriology, Bu Ali Sina University). First, bacteria were grown to obtain 1 × 10^6^ CFU mL^−1^. Then, micro broth dilution assays were performed as described for Anti proliferative Assays, but in nutrient broth (NB) medium. The experimental plates were incubated at 28°C for 16 h for Plant pathogenic bacteria and 37°C for 16 h for Human pathogenic bacteria. The growth of target bacteria was observed and compared with the control to determine the MIC and the minimum bactericidal concentration (MBC). The experiments were performed in triplicate. Upon obtaining the required data, the metabolite concentration required for 80% of *in vitro* inhibition was presented as IC_80_ value.

### TLC bioautography of the *T. koningiopsis* VM115

According to the information provided in Tables 3–**5** between methanol and ethyl acetate extracts, crude ethyl acetate extract of *T. koningiopsis* VM115 isolate was used for TLC and GC-MS analysis. The ethyl acetate extract of each isolated fungus was introduced to silica gel plate (10 × 5 cm) by adding a fluorescent indicator, which was developed by placing it in a paper-linked filter in a glass chromatography tank containing 50 ml of an evenly mixed solution of CHCl_3_/ MeOH (v/v, 10: 1). As soon as the solvent height was observed to reach 9.5 cm, the plates were removed from the tank. The presence of UV-absorbing compounds was corroborated after seeing dark spots on the dried plates under a UV radiation of 254 nm. The spots were marked with a pencil. Then, using an aerosol spray, a suspension of 10^6^ *P. oryzae* spores per ml was gently and evenly sprayed onto the plates to make it turn translucent. In a biological hood, the fungal suspension was prepared and sprayed on to the plates using gloves. Washing of the spray bottle with 70% ethanol and sterile distilled water was followed prior to use. The plates were placed in plastic bags in a light box at 28 ± 1°C for a 12-h photoperiod. Within 1 week, the plates were photographed under UV at 254 nm and the inhibition zones were observed as white circular areas of reduced densities or as the fungus non-growth area.

**Table 3:** Antiproliferative activities of (EAC and MET) metabolites from endophytic *Trichoderma* species against the conidia of *Pyricularia oryzae* the observations were averages of 4–6 assays.

**Isolate**	**The final concentrations of Methanol and Ethyl acetate (in** μ**g ml**^**−1**^**)**											
	**250.0**		**125.0**		**62.5**		**31.2**		**15.6**		**7.8**	
	**EAC**	**MET**	**EAC**	**MET**	**EAC**	**MET**	**EAC**	**MET**	**EAC**	**MET**	**EAC**	**MET**
*T. koningiopsis* VM115	^*^	^*^	^*^	^*^	^*^	^*^	^*^	+++	++	+	+	–
*T. longibrachiatum* VM99	^*^	^*^	^*^	^*^	^*^	^*^	^*^	+++	+++	+	–	–
*T. brevicompactum* VM102	^*^	^*^	^*^	^*^	^*^	^*^	+++	+++	++	–	–	–
*T. longibrachiatum* VH104	^*^	^*^	^*^	^*^	^*^	+++	+++	++	++	–	–	–
*T. brevicompactum* VM98	^*^	^*^	^*^	^*^	+++	+++	++	++	+	–	–	–
*T. longibrachiatum* VM111	^*^	^*^	^*^	+++	+++	+++	+++	++	+	–	–	–
*T. asperellum* VM 100	^*^	^*^	^*^	+++	+++	++	++	++	+	–	–	–

*The observations were averages of 4–6 assays*.

* The P. oryzae conidial germination was completely inhibited;

+++Strong growth inhibition of germ tube (≤1/3 of control);

++ Moderate Growth inhibition of germ tube (1/3–2/3 of control);

+Low Growth inhibition of germ tube (≥2/3 but less than control);

*–Not inhibited (as control)*.

*MET, Methanol extract; EAC, Ethyl acetate extract; T, Trichoderma*.

### Extraction and fractionation of *T. koningiopsis* VM115 culture

After obtained following the process described above, a culture filtrate of *T. koningiopsis* VM115 was extracted with ethyl acetate. A residue (20.0 g) was produced in vacuo evaporation of the organic solvent from the extract and dissolved in a lower amount (10 ml) of MeOH around 45°C. The resultant liquor was stored at −10°C overnight, followed by filtration to remove any waxy materials such as the precipitate. MeOH removal under reduced pressure provided a brown lump, and MeOH was added to it until it was completely dissolved. Acetone was added to the MeOH solution drop by drop with acetone; finally at 15% (v/v), it was kept overnight below −10°C to precipitate salts and saccharides. The obtained filtrate was concentrated in vacuo to render a residue (13 g), which was then exposed to column chromatography over Si gel column (100 g, 200–300 mesh) eluting with petroleum ether-acetone mixtures (v/v, 1: 0, 20: 1, 10: 1, 5: 1, 3: 1, 0: 1, 800 ml each) to provide five fractions. Fr.2 (1.9 g)active to the test fungi by TLC bioautography was rechromatographed over Si gel column only (50 g, 200–300 mesh), eluting with CHCl_3_-MeOH gradient (v/v, 100:1, 50:1, 20:1, 10:1, 400 ml each). Fr2 was separated further on a Sephadex LH-20 column, eluting with acetone to achieve eight sub fractions Fr2 (1–8). On a silica gel column (20 g), active Fr2 (3) was then purified and eluted with petroleum ether/ethyl acetate (20:1, v/v) to yield 1.045 g of active compound (1). Compound 1 was identified by spectral analyses including MS, ^1^H-NMR, ^13^C-NMR (Liu et al., 2006).

### Antifungal assay of *T. koningiopsis* VM115 compound

Antifungal activities of compound 1 were examined *in vitro* by testing *P. oryzae*, Botrytis cinera and *Aspergillus fumigatus* through the method described in the literature (Barchiesi et al., 2000) based on ketoconazole co-assay as a positive reference. Each compound was isolated in triplicate.

### GC-MS conditions of *T. koningiopsis* VM115

An Agilent technologies 7890A gas chromatograph connected to a 5975 Cinert MSD was used to identify volatile and semi-volatile secondary metabolites from the crude extract of fungi isolate. Also, an HP-5MS fused silica capillary column was applied (Hewlett-Packard, 30 m × 0.25 mm i.d. 0.25 μm film, cross-linked to 5% phenyl methyl siloxane stationary phase). The entire system was checked using Chemstation software (Hewlett-Packard, version A.01.01). Electron impact mass spectra were recorded at 70 eV, and ultra-high pure HAE (99.999%) gas was used as the carrier gas at flow rate of 1 Ml min^−1^. The injection volume was found to be 1 μL, and all injections were done in a split-less mode. The injector and detector temperature settings were 250 and 280°C, respectively. Column oven temperature was set initially at 50°C for 5 min, then raised to 260°C (ramp: 4°C/min) and held for 5 min. The database of the National Institute of Standards and Technology (NIST) was applied to interpret mass spectrum of GC-MS with more than 62,000 patterns. Using the data obtained from NIST05 (National Institute of Standards and Technology, US), WILEY 8, and FFNSC1.3 (Flavor and Fragrance Natural and Synthetic Compounds) libraries, the existing bioactive compounds in the extracts were identified through a comparison of mass spectra. The molecular weights and structural components of the test materials were determined. Ultimately, most of the compounds of this fungus were identified and verified by comparing the standard GC/MS data with those of the fungal products.

### Statistical analysis

SAS (Ver. 9.1), statistical software was used to compare the means through the test of Least Significant Difference (LSD). The differences between the varied treatments were specified at 5% level (*P* = 0.05).

## Results

### Host and fungi identity and phylogeny of *Trichoderma* species isolates

**Figure 1:**
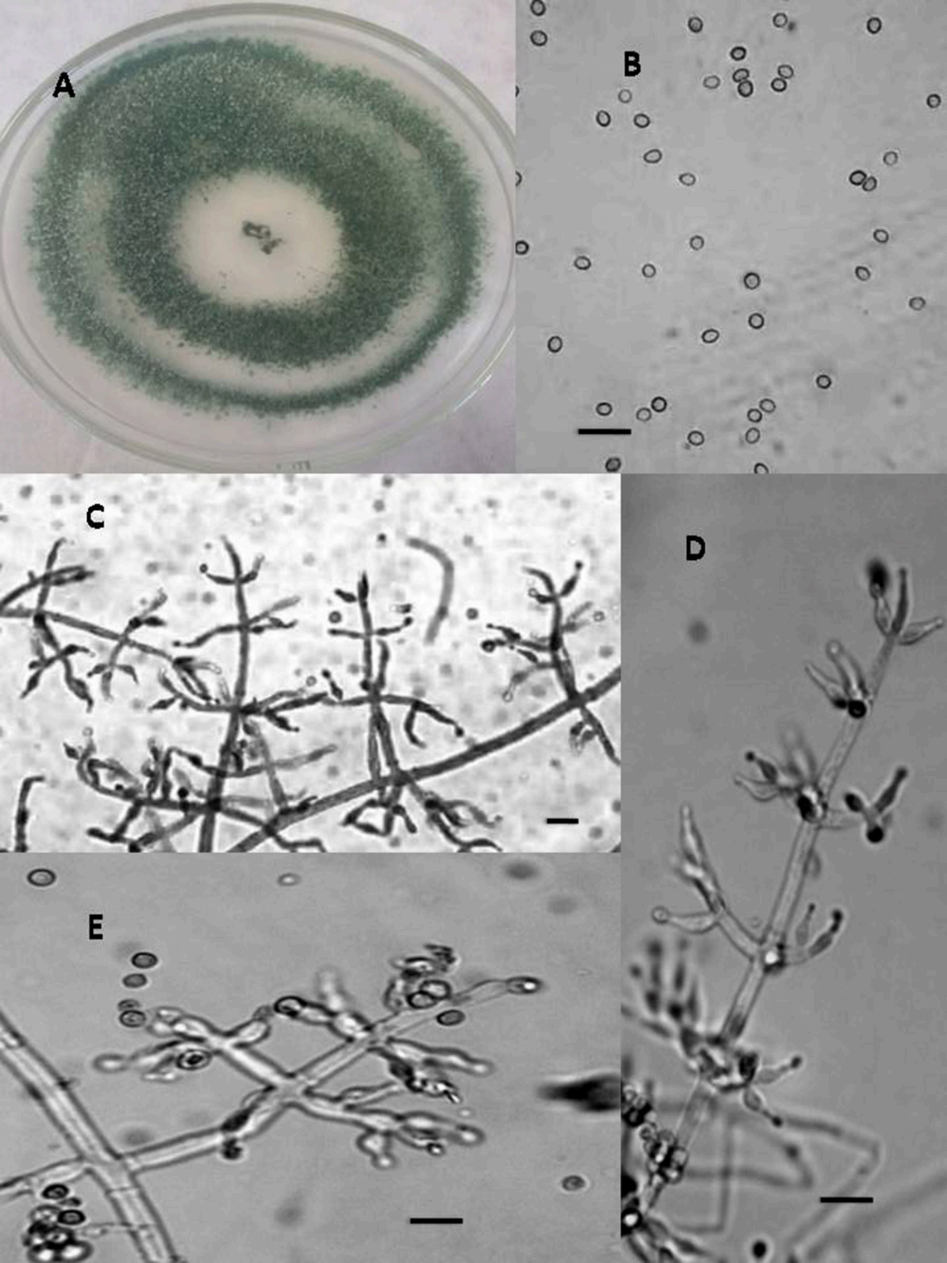
Morphology of *T. asperellum* Colony appearance on PCA (Potato Carrot Agar) **(A)**; conidia **(B)**; conidiophores **(C–E)**, Scale Bar 10 μm.

**Figure 2:**
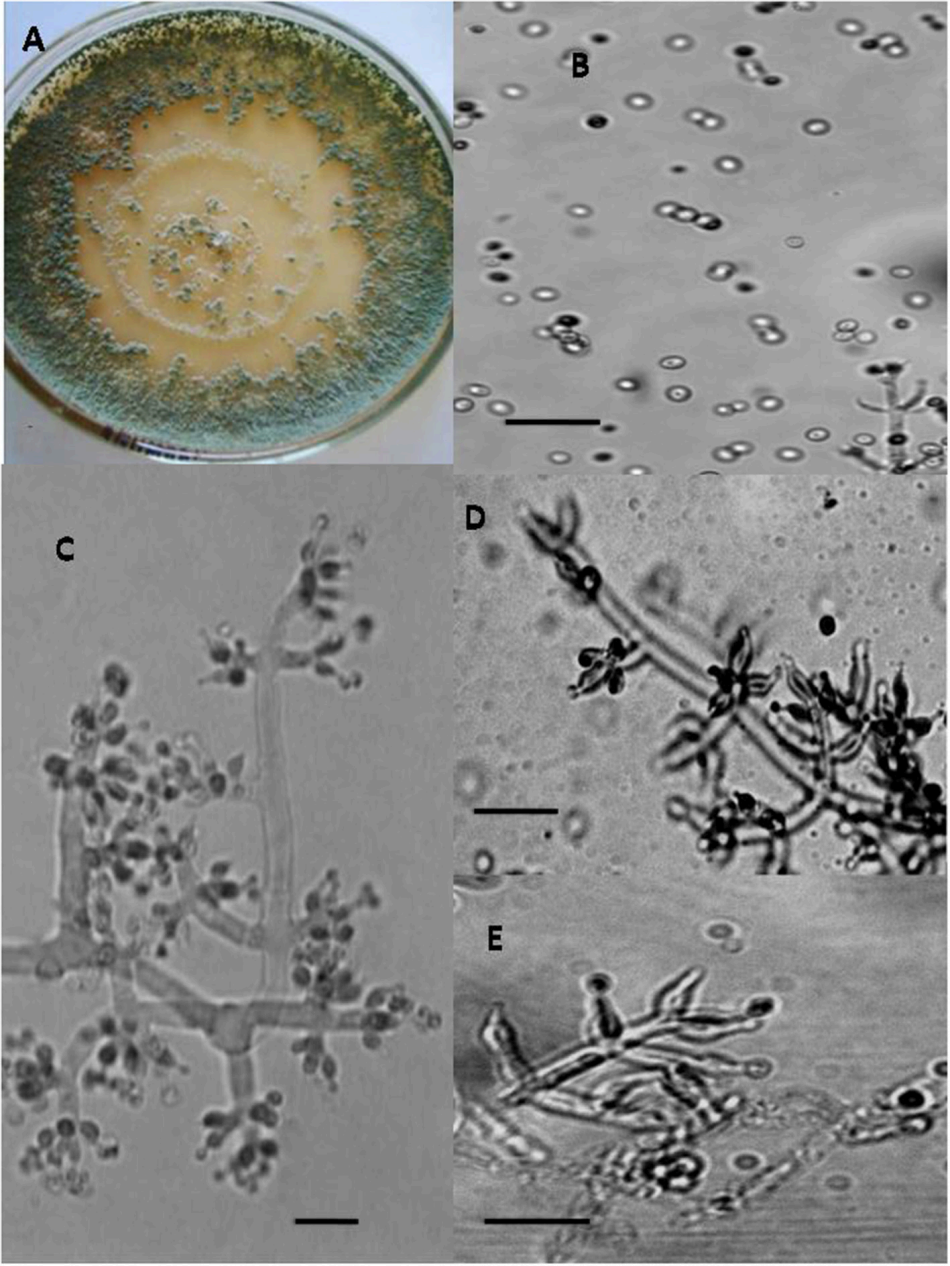
Morphology of *T. brevicompactum* Colony appearance on PCA (Potato Carrot Agar) **(A)**; conidia **(B)**; conidiophores **(C–E)**, Scale Bar 10 μm.

**Figure 3:**
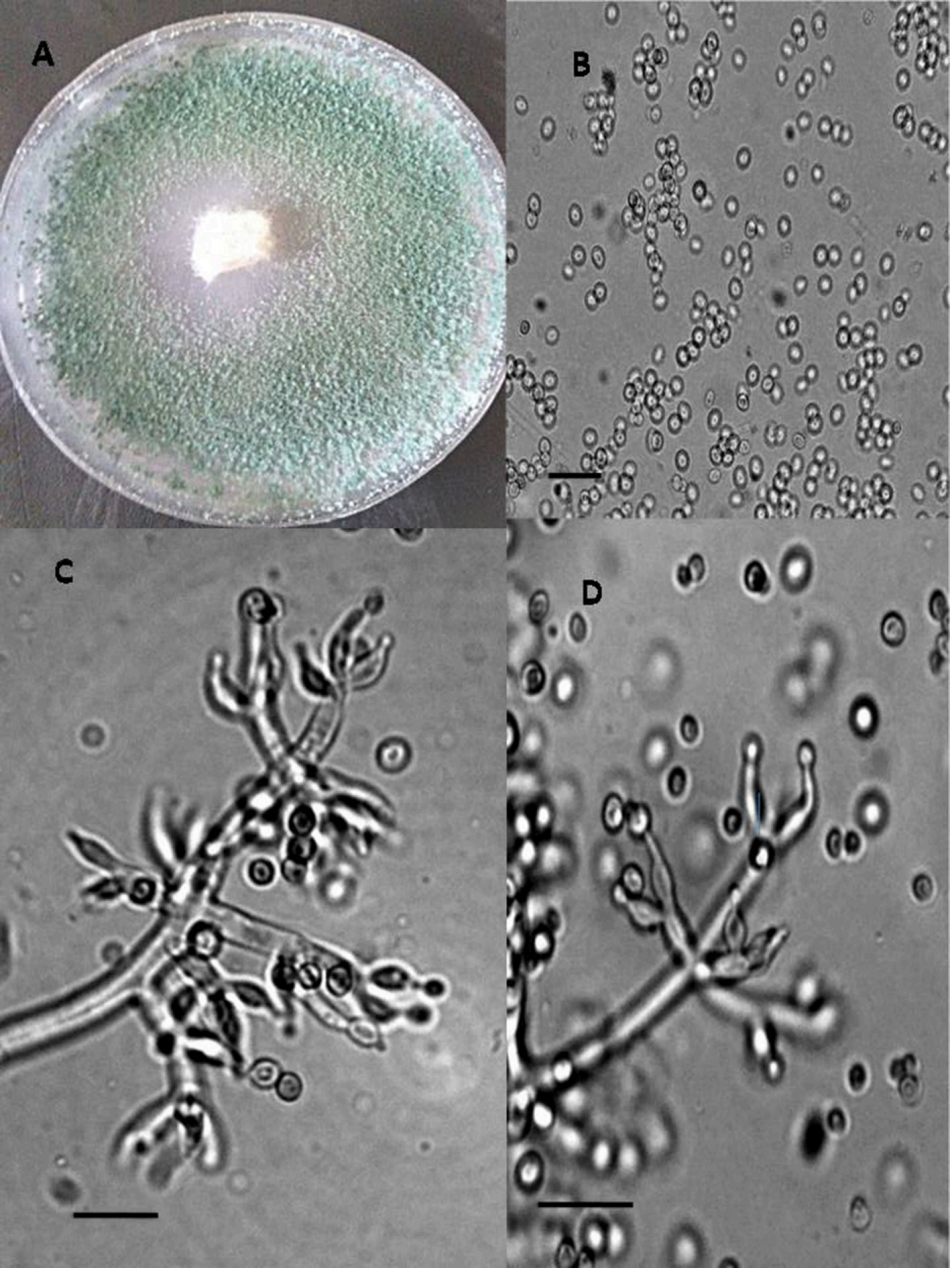
Morphology of *T. koningiopsis* Colony appearance on PCA (Potato Carrot Agar) **(A)**; conidia **(B)**; conidiophores **(C,D)**, Scale Bar 10 μm.

**Figure 4:**
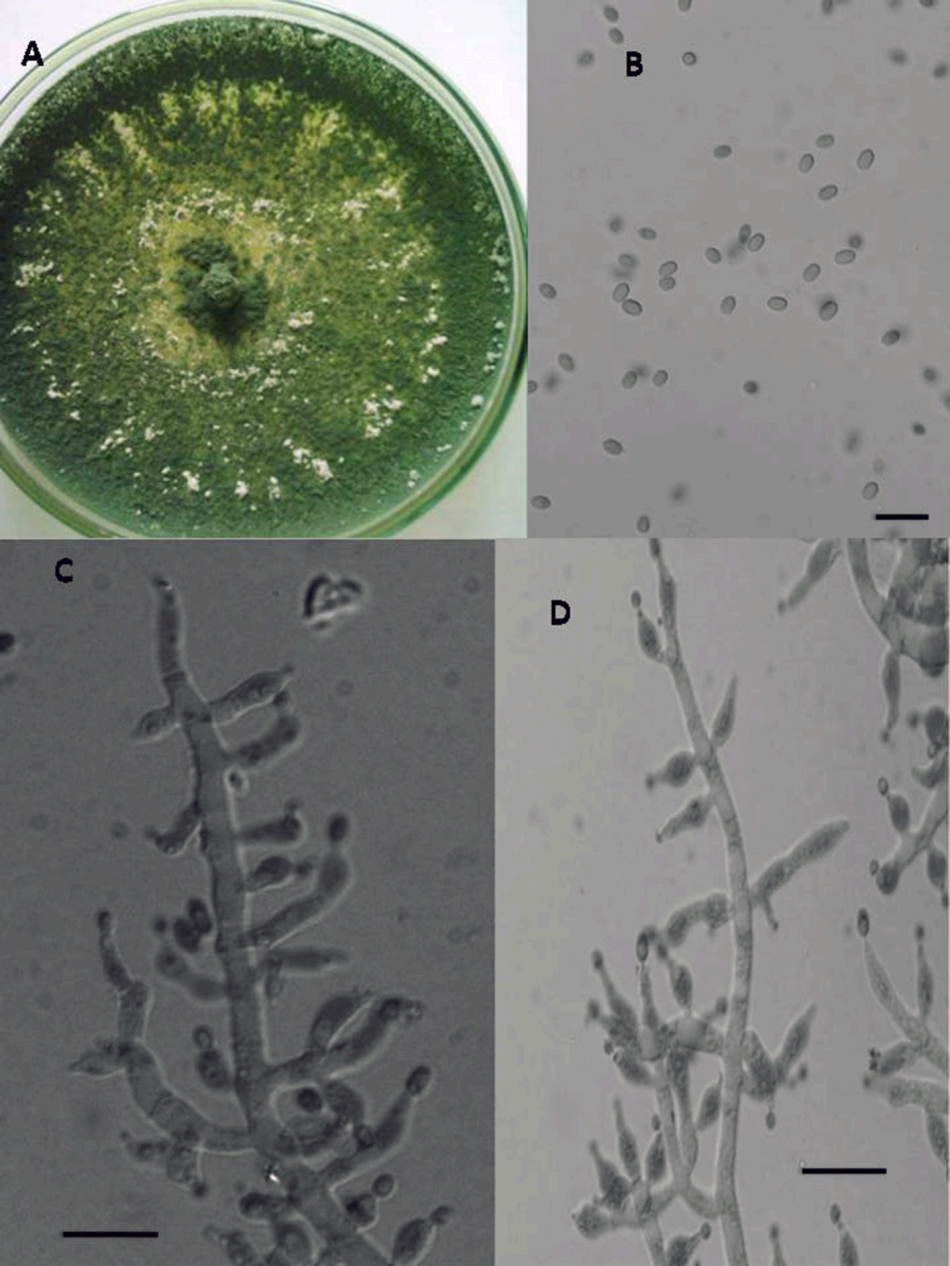
Morphology of *T. longibrachiatum* Colony appearance on PCA (Potato Carrot Agar) **(A)**; conidia **(B)**; conidiophores **(C,D)**, Scale Bar 10 μm.

**Figure 5:**
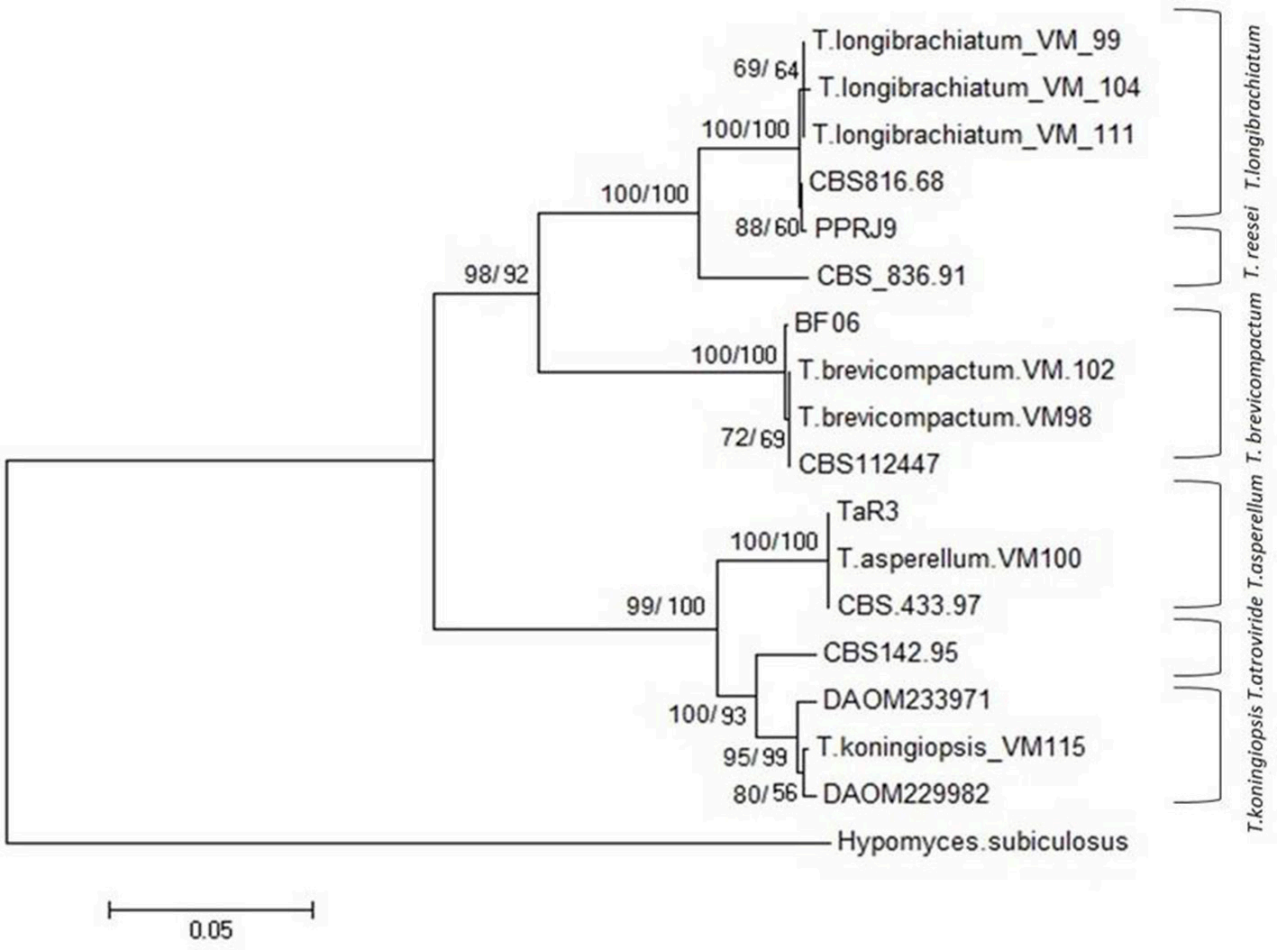
NJ tree based on combined dataset of ITS and EF1-α sequences. Bootstrap values. 50% (1,000 replicates) shown above branches [those of parsimony analysis (1,000 replicates) are shown on the right side Slash]. Scale bar indicates nucleotide substitution in NJ analysis. *Hypomyces subiculosus* is outgroup. T*, Trichoderma*.

In total, seven endophytic *Trichoderma* fungal isolates were recovered from six different locations from 500 plant specimens from healthy above ground tissue (leaf, stem) of the *Vinca major, Vinca herbacea*, and *Vinca minor (Apocynaceae* family) (Table 1). Endophytes were classified according to morphological traits using the key in Gams and Bissett (1998) (Figures 1–4). By combining the 2 unlinked regions of the genes including ITS/ EF1-α, the phylogenetic analyses were conducted. The internal nodes were observed to highly support the phylogenies that resulted from the stable and reproducible ITS/ EF1-α. Production of the trees with similar topologies was represented by the individual congruent datasets since no significant divergence (*P* = 0.48) was seen through the partition homogeneity test in PAUP 4.0b10 (Swofford, 2003). Therefore, ITS and EF1-α datasets were combined for analysis. The combined ITS and EF1-α sequences for 17 ingroup and 1 outgroup taxa contained 1,574 characters including alignment gaps, of which 334 characters were excluded, 482 were constant, 557 were variable and parsimony-uninformative and 535 were parsimony-informative. A heuristic search of the remaining 535 parsimony-informative characters resulted in a single most parsimonious tree of 426 steps (CI = 0.76, HI = 0.23, RI = 0.86). Seven isolates from four provinces were sequenced for ITS and tef1 and species were identified according to a combination of morphologic and genotypic characters. The identification details of these isolations and their origins are shown in Table 1, in which 4 species are recognized: *Trichoderma asperellum, Trichoderma brevicompactum, Trichoderma koningiopsis, Trichoderma longibrachiatum*. Results of the phylogenetic analysis based on ITS and EF1-α sequences are shown in Figure 5. Overall, four fungal species were morphologically identified by ITS and EF1-α sequence analyses.

### Anti-proliferative bioactivity of methanol and ethyl acetate extracts

To primarily screen the antitumor activities, *P. oryzae* fungus was used as a model target (Kobayashi et al., 1996; Dong et al., 2008; Xu et al., 2009). Accordingly, conidial germination and development of germ tube from *P. oryzae* was adapted for evaluating anti-proliferative activity of methanol and ethyl acetate extracts *Trichoderma* metabolites. The results presented in Table 3 demonstrate significant bioactivity for all *Apocynaceae* endophytic *Trichoderma* isolates. These findings indicate that the ethyl acetate extracts from endophytic *Trichoderma* isolates showed higher anti-proliferative effects compared to methanol extracts. Notably, the isolate *T. koningiopsis* VM115 showed the most significant bioactivity among all isolates. *T. koningiopsis* VM115 showed that conidial germination was completely inhibited at 250–31.2 μg mL^−1^ and inhibition of germ tube elongation at a concentration of 31.2–7.8 μg mL^−1^.

### Cell viability assay

**Figure 6:**
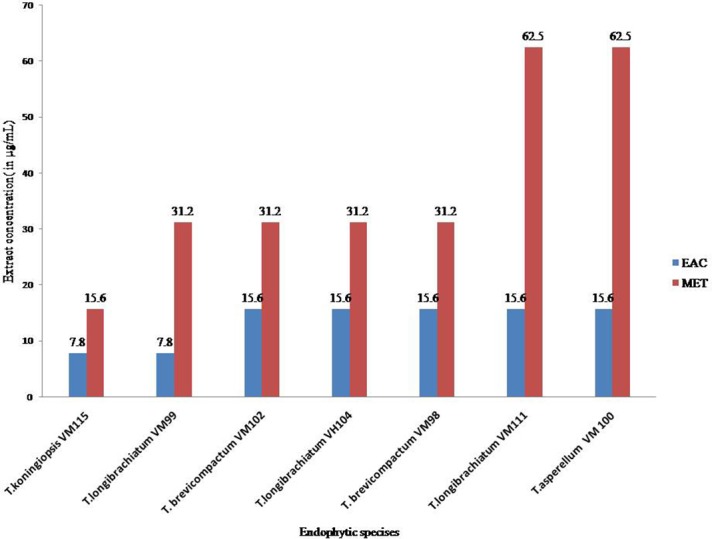
Cell viability assays of (EAC and MET) metabolites from endophytic species. Data (significant at P B 0.05) were obtained from three replicates. Data are reported as IC50 values. MET, Methanol extract; EAC, Ethyl acetate extract.

The results of the Cell viability of the extracts of *Trichoderma* isolates are shown in Figure 6. All *Trichoderma* isolates metabolites exhibited significant activity against *P. oryzae* conidia after treatment with ethyl acetate extracts and methanol extracts with an IC_50_ value at a range of 7.8–31.2 μg mL^−1^ and 15.6–62.5 μg mL^−1^, respectively. Also ethyl acetate extracts from all endophytic isolates showed higher cytotoxic effects compared to methanol extracts. In total, ethyl acetate extracts of *T. koningiopsis* VM115 isolate was the most cytotoxic at 7.8 μg mL^−1^. However, no inhibitions were found in the untreated *P. oryzae* conidial suspension. The results of cell viability assay and antiproliferative activity indicated that significant cell growth inhibition for all *Apocynaceae* endophytic *Trichoderma* isolates. The data elicited strong likelihood of cell growth inhibition and cytotoxic effects by *T. koningiopsis* VM115 isolate.

### Bacteriostatic and bactericidal bioactivity of fungal crude extract

All endophytic isolates were screened for antibacterial activity using two human pathogens (*S. aureus* PTCC and *E. coli* PTCC) and two plant pathogens (*Ral. solanacearum* and *Cl. michiganensis*) as targets. All *Trichoderma* isolate metabolites showed bacteriostatic and bactericidal activity against the two Gram-negative and two Gram-positive bacteria targets (Table 4). Ethyl acetate extracts were bacteriostatic with an IC_80_ value at a range of 7.8–15.6 μg mL^−1^ and bactericidal with an IC_80_ value at a range of 15.6–62.5 μg mL^−1^. The methanol extracts were bacteriostatic with an IC_80_ value at a range of 7.8–62.5 μg mL^−1^ and bactericidal with an IC_80_ value at a range of 31.2–62.5 μg mL^−1^. Also, *T. koningiopsis* VM115 and *T. longibrachiatum* VM 99 were more influential against Gram-negative and Gram-positive bacteria. Ethyl acetate extract had higher-level activity than other extracts, compared with bioactivity between methanol and ethyl acetate extract.

**Table 4:** The antibacterial activities of (EAC AND MET) metabolites from endophytic species.

**Isolate**	**Target bacteria**	**Extract concentration (in** μ**g ml**^**−1**^**)**			
		**MIC^a^**		**MBC^b^**	
		**EAC**	**MET**	**EAC**	**MET**
*T. koningiopsis* VM115	*S. aureus*	7.8	7.8	15.6	31.2
	*Ral. solanacearum*	7.8	7.8	15.6	31.2
	*E. coli*	7.8	15.6	31.2	62.5
	*C. michiganensis*	7.8	15.6	31.2	62.5
*T. longibrachiatum* VM99	*S. aureus*	7.8	7.8	15.6	31.2
	*Ral. solanacearum*	7.8	7.8	15.6	31.2
	*E. coli*	7.8	15.6	31.2	62.5
	*C. michiganensis*	15.6	15.6	31.2	62.5
*T. brevicompactum* VM102	*S. aureus*	7.8	15.6	15.6	31.2
	*Ral. solanacearum*	7.8	15.6	15.6	31.2
	*E. coli*	15.6	31.2	31.2	62.5
	*C. michiganensis*	15.6	31.2	31.2	62.5
*T. longibrachiatum* VH104	*S. aureus*	7.8	15.6	31.2	31.2
	*Ral. solanacearum*	7.8	31.2	31.2	31.2
	*E. coli*	15.6	31.2	15.6	31.2
	*C. michiganensis*	15.6	15.6	62.5	62.5
*T. brevicompactum* VM98	*S. aureus*	15.6	31.2	31.2	62.5
	*Ral. solanacearum*	15.6	31.2	31.2	62.5
	*E. coli*	15.6	31.2	62.5	62.5
	*C. michiganensis*	15.6	31.2	62.5	62.5
*T. longibrachiatum* VM111	*S. aureus*	7.8	15.6	31.2	62.5
	*Ral. solanacearum*	7.8	15.6	62.5	62.5
	*E. coli*	15.6	31.2	62.5	62.5
	*C. michiganensis*	15.6	31.2	62.5	62.5
*T. asperellum* VM 100	*S. aureus*	15.6	31.2	62.5	62.5
	*Ral. solanacearum*	15.6	31.2	62.5	62.5
	*E. coli*	15.6	31.2	62.5	125
	*C. michiganensis*	15.6	31.2	62.5	125

*Data (significant at P B 0.05) were obtained from three replicates. Data are reported as IC80 values*.

a *Minimum inhibitory concentration*.

b *Minimum bactericidal concentration*.

*MET, Methanol extract; EAC, Ethyl acetate extract*.

### Identifications of metabolites

**Figure 7:**
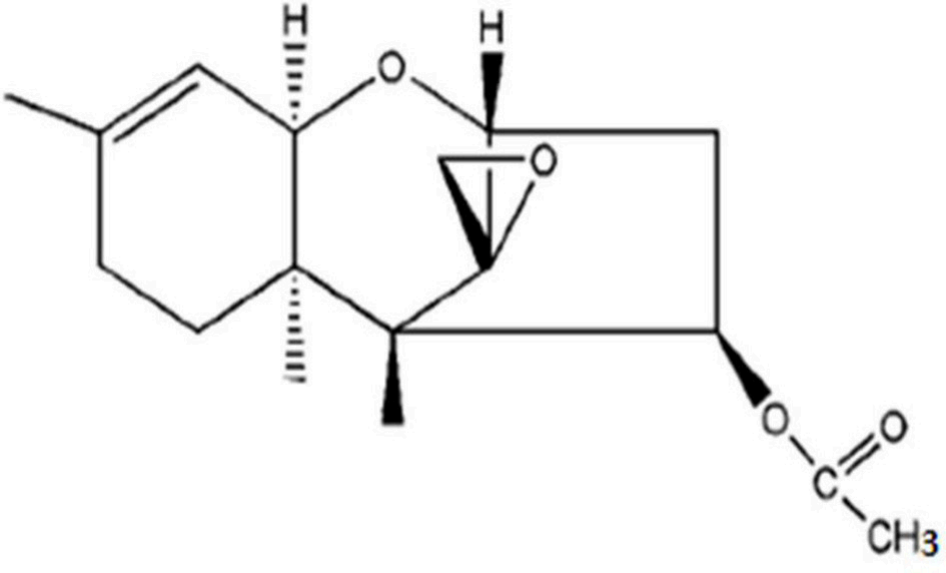
Trichodermin (4*b*-acetoxy-12, 13-epoxytrichothec-9-ene).

According to the information provided in Tables 3–5 between ethyl acetate and methanol extracts, crude ethyl acetate extract of *T. koningiopsis* VM115 isolate was used for TLC and GC-MS analysis. The one known antifungal metabolite purified from the ethyl acetate extract *T. koningiopsis* VM115 was identified as compound 1 (Godtfredsen and Vangedal, 1964) via spectral analyses, such as MS, ^1^H-NMR, ^13^C-NMR (Figure 7).

**Table 5:** The MIC values of compounds 1 and 2 (in μg ml^−1^).

**Compounds**	***P. oryzae***	***B. cinera***	***A. fumigatus***
4b-hydroxy-12, 13-epoxytrichothec-9-ene	31.2	31.2	31.2
Ketoconazole^*^	62.5	31.2	62.5

* *Ketoconazole was co-assayed as a positive control*.

*Data (significant at P B 0.05) were obtained from three replicates*.

Trichodermin (4*b*-acetoxy-12, 13-epoxytrichothec-9-ene) = colorless oil. ^1^H NMR (CDCl3, 400 MHz) δ: 5.48 (1H, dd, J = 7.8, 3.6 Hz, H-4), 5.31 (1H, d, J = 5.4 Hz, H-10), 3.71 (1H, d, J = 5.2 Hz, H-2), 3.50 (1H, d, J = 5.4 Hz, H-11), 3.02 (1H, d, J = 4.0 Hz, H-13a), 2.73 (2H, m, H-13b, H-3a), 2.46 (1H, dd, J = 15.4, 7.8 Hz, H-3b), 1.81–1.97 (3H, m, H-7a, H-8a, H-8b), 1.62 (3H, s, H-16), 1.33 (1H, brd, J = 12.1 Hz, H-7b), 0.83 (3H, s, H-15), 0.61 (3H, s, H-14). ^13^C-NMR (CDCl3,100 MHz) δ: 170.6 (s, C-1'), 139.8 (s, C-9), 118.3 (d, C-10), 78.8 (d, C-2), 74.7 (d, C-4), 70.1 (d, C-11), 65.2 (s, C-12), 48.6 (s, C-5), 47.5 (t, C-13), 40.1 (s, C-6), 36.3 (t, C-3), 27.7 (t, C-8), 24.1 (t, C-7), 23.0 (q, C-16), 20.9 (q, C-2'), 15.7 (q, C-15), 5.5 (q, C-14).

ESI-MS: 315 [M + Na]^+^, 607 [2 M + Na]^+^, 251 (M+H)^+^.

### Antifungal assay of trichodermin

*T. koningiopsis* VM115 compound was bio assayed against pathogenic and model fungi *B. cinera, A. fumigatus*, and *P. oryzae* by the serial dilution method (Table 5). Having an MIC close to that of ketoconazole as a positive control, compound 1 was seen to be involved in a strong bioactivity against the test fungi.

### GC-MS analysis

Crude ethyl acetate extract of *T. koningiopsis* VM115 isolate was used for GC-MS analysis. The mass spectra of GC-MS-MS were interpreted by the database of National Institute of Standards and Technology (NIST) with more 62,000 patterns. The chromatogram anticipated the presence of many compounds and they were identified according to peak area, the retention time, and molecular formula. The retention time and abundance of the compounds under the described conditions in GC-MS section are shown in Table 6; the identification of these metabolites revealed that endophytic *T. koningiopsis* VM115 has the capacity to produce bioactive compounds. Collectively, each of the seven classes of volatile compounds was generated by *T. koningiopsis* VM115 [alcohols, esters, monoterpene, pyrones (lactones), acids, furanes, and lipids] (Table 6).The most abundant compound was 6-pentyl-alpha-pyrone (6-PP) with a 20.442 min retention time, based on the total area of the GC analysis, originally specified by Collins and Halim (1972), and indicated as one of the important bioactive compounds from *T. harzianum* and *T. koningii* species as reviewed by Hanson (2005).

**Table 6:** GC/MS analysis of the volatile compounds produced by *T. koningiopsis* VM115.

**Peak**	**Chemical name**	**Chemical name**	**Std**	**RT (min)**	**Abundance (%)**	**Peak**	**Chemical name**	**Chemical name**	**Std**	**RT (min)**	**Abundance (%)**
1	Butanoic acid, Butyl ester	C8H16O2		5.325	78.57	12	1-pyrrolidinamine	C4H10N2		18.317	70
2	Butyrolactone	C_4_H_6_O_2_		6.478	82	13	Isosorbide	C6H10O4		18.833	70.62
3	N,N-dimethyl-formamide	C3H7NO	^*^	6.500	82	14	Hexadecanoic acid	C20H40O2		19.058	85
4	Sulfurous acid, octyl 2-pentyl ester	C13H28O3S		7.099	72.80	15	3H-pyrazol-3-one	C6H10N2O		19.992	84.32
5	Ethanoic acid	C2H4O2	^*^	8.171	80	16	2H-pyran-2-one	C6H10O3		20.442	85
6	2,4-dimethylbenzaldehyde	C9H10O		12.575	78.97	17	2-propenyl ester, Pentanoic acid	C8H14O2		20.600	84
7	2-butoxyethyl acetate	C8H16O3		13.842	74	18	2,6-dimethyl-naphthalene	C12H12		25.883	78
8	21 b phenylethyl alcohol C8H10O	C3H6O2		14.175	79	19	Hexadecane	C_16_H_34_	^*^	30.334	97
9	1-hydroxy-2- propanone	C6H6O		14.983	75.79	20	Heptadecane (C17)	C17H36		30.458	77.72
10	4H-pyran-4-one	C6H8O4	^*^	17.692	70.80	21	Phthalic acid, 5-methoxy-3-methylpentyl propyl ester	C20H37O5		52.547	71.17
11	3,5-bis(1,1-dimethylethyl)phenol	C14H22O		18.083	75	22	2-Octene(mixed cis, trans isomers)	C_8_H_16_		55	73

* *Standard authentic compounds having the same RT and MS as the fungal product*.

*Only compounds with quality match scores >70 are listed. Details of VOC extraction and GC/MS analysis are in Methods section*.

## Discussion

It has become evident in the last two decades that all healthy plants on earth harbor endophytic microorganisms (Strobel and Daisy, 2003). Much investigation has been done to explore biodiversity and bioactivity of fungal and bacterial endophytic microorganisms associated with numerous host plant species (Strobel and Daisy, 2003; Aly et al., 2010; Kusari et al., 2012, 2013). The present study investigated biodiversity and bioactivity of *Trichoderma* endophytic species in plants of the *Vinca* plants growing in Iran. Tests were conducted on 7 isolates from five provinces in Iran; Esfahan, Mazandaran, Hamedan, and Tehran. A wide variety of species was demonstrated by the results: *T. asperellum* (1 isolate), *T. brevicompactum* (2), *T. koningiopsis* (1), *and T. longibrachiatum* (3). Endophytic *T. longibrachiatum* showed ubiquitous dispersion in all sampling locations and on all three plants, and *T. brevicompactum* was isolated from *V. minor* and *V. major* but *T. asperellum*, and *T. koningiopsis* isolates were isolated from only one of the plants (Table 1). Our findings provide further indication that endophytic *Trichoderma* fungal species isolates exhibit significant anti-proliferative, cytotoxic and antimicrobial activity. Moreover, in general, ethyl acetate extract metabolite from endophytic isolates showed higher cytotoxic and antibacterial activity than methanol extracts. Also, varied secondary metabolites with robust antifungal and antibiotic activities are produced by *Trichoderma* species (Siddiquee et al., 2012). Moreover, amongst all, bioactivity of ethyl acetate and methanol extract metabolites from *T. koningiopsis* VM115 were superior compared to the others. This was demonstrated against *P. oryzae* (Table 3 and Figure 6). Abdulmyanova et al. (2015) evaluated ethyl acetate (EtAc) extracts of endophytes from *V. minor* and *V. erecta*. They demonstrated that these extracts have potential cytotoxic activity on three cancer cells, indicating the presence of cytotoxic compounds in these extracts that were exerted against the survival and growth of the model bacteria (Table 4). This result might indicate the presence of a series of compounds in *T. koningiopsis* VM115 that make it compete with or attack antagonist cells in a mixed population within environmental niches. This may suggest that the endophytic species of *Trichoderma* have evolutionary, selective, and protective roles when living inside their host plants.

Trichothecenes comprise a group of sesquiterpenes, which are structurally characterized with a 12, 13-epoxy-trichothec-9-ene moiety. Interestingly, the nucleus of this sesquiterpene has been reported to appear in fungal cultures containing *Fusarium, Myrothecium, Stachyobotrs*, and *Trichoderma* (Ueno, 1985; Abbas et al., 2002; Liu et al., 2006). Biologically, it has been reported that macrocyclic trichothecenes have antimalarial (Zhang et al., 2002), antiviral (Garcia et al., 2002), antifungal and antibacterial (Wagenaar and Clardy, 2001), and insecticidal activities (Cole and Cox, 1981) besides being phytotoxic and cytotoxic (Abbas et al., 2002) to animals. In the current research, *T. koningiopsis* VM115 as a trichothecene was seen to have antifungal materials in its ethyl acetate extract with a comparable MIC with that of ketoconazole as a positive control (Table 5). This compound with fungicidal activity-Trichodermin (4b-hydroxy-12, 13-epoxytrichothec-9-ene) (Godtfredsen and Vangedal, 1964; Yang et al., 2010) as a member of 4β-aceoxy-12, 13-epoxytrichothecene family A belongs to the class of trichothecenes, a group of sesquiterpene toxins. The mechanism of action for this class of toxins is mainly protein biosynthesis inhibition by preventing peptidyl transferase activity, although it was initially thought to be potentially useful in anticancer therapeutics. Isolation of Trichodermin from a few species of *Trichoderma*, such as *T. brevicompactum* and *T. viride, T. Longibrachiatum*, and *T. harzianum* has been carried out and it includes the first reported Trichodermin production of *T. koningiopsis* (Godtfredsen and Vangedal, 1964; Watts et al., 1988; Nielsen et al., 1998, 2005; Reino et al., 2008; Yang et al., 2010; Tijerino et al., 2011).

A multifaceted interaction occurs between filamentous fungi and their living environments mainly with the help of volatile metabolites. Secondary metabolism of fungi may have a role in plant defense, when the mixture of volatile compounds was broken down into several classes of compound, the same inhibitory effects were not achieved. This suggests that it is the suite of volatile compounds that contributes to antifungal activity (Strobel et al., 2001; Strobel, 2003). Along with providing defense against pathogens of their host; certain endophytic fungi may aid plant survival in certain habitats. No volatile compounds have been identified to be solely involved in such bio-control activities though some are known to be associated with the antagonistic ability of *Trichoderma* species (Siddiquee et al., 2012). The most abundant metabolite originally characterized by Collins and Halim (1972) was identified to be 6-pentyl-alpha-pyronein this research.6-pentyl-2H-pyran-2-one (6-pentyl–prone) as a metabolite is responsible for the coconut aroma released from axenic ally grown colonies. It is commonly purified from the culture filtrates of different *Trichoderma* species, such as *T. viride, T. atroviride, T. harzianum*, and *T. koningii*. Both *in vivo* and *in vitro* antifungal activities against several plant pathogenic fungi have been shown by 6PP. Moreover, biosynthesis of this metabolite has been shown to have a strong relationship with the bio-control ability of the producing microbe (Kobayashi et al., 1996, 2004; Vinale et al., 2014). Many important components were produced by *T. koningiopsis* VM115. Fatty acids and hydrocarbons were produced by *T. koningiopsis* VM115. Fatty acids are organic acids with antibacterial and antifungal activities (Pohl et al., 2011). Ethanoic acid, hexadecanoic acid, and butanoic acid (as an unsaturated fatty acid) produced by *T. koningiopsis* VM115. These compounds were reported in the *T. viride* by Gershon and Shanks (1978). Hydrocarbons such as hexadecane were also produced. Previously, butyrolactone was isolated from *Aspergillus terreus* (Arai et al., 1982). Studies of endophytic fungal diversity have mainly determined relationships among endophytic fungi as well as their host plants, by looking for natural bioactive compounds obtained from the endophytic fungi. Endophytic fungi were shown to effectively yield many vital bioactive compounds with antimicrobial, insecticidal, cytotoxic, antioxidant, and anticancer activities. *Trichoderma* sp. is the most prominent genus among the mycoflora. All of the isolates of endophytic fungi exhibited significant antiproliferativeand antimicrobial activity on selected test organisms. TLC guided fractionation and MS, ^1^H-NMR, ^13^C-NMR data showed that trichodermin compound was isolated from *T. koningiopsis* VM115, which demonstrated strong fungicidal effects against selected test organisms through *in vitro* antifungal tests. Also, GC-MS analysis determined six classes of volatile compound produced by *T. koningiopsis* VM115. 6-n-pentyl-6H-pyran-2-one (6PP) was identified as one of the most abundant metabolites in this research. These results indicate that the fungal endophytes from *Vinca* plants had antibacterial and cytotoxic activities; evidence that endophytes are a good source of biological activity and compounds. These are very promising for application in agriculture and medicine. It should be significant for us to screen for antibacterial activities on fungal endophytes from *Vinca* plants. Endophytes were found to be a good source for compounds with biological activities as evidenced by the cytotoxic and antibacterial activities of the fungal endophytes obtained from *Apocynaceae*. Therefore, *Trichoderma* endophytic species are promising sources of new and natural bioactive metabolites that provide a great potential for further research.

## Author contributions

Both authors carried out the molecular genetic studies, participated in the sequence alignment and drafted the manuscript. Both authors carried out the antiproliferative, cytotoxic, and antimicrobial activities and TLC, GC-MS, MS, ^1^H-NMR, ^13^C-NMR analysis. Both authors participated in the sequence alignment. Both authors participated in the design of the study and performed the statistical analysis. Both authors conceived of the study, and participated in its design and coordination. All authors read and approved the final manuscript.

### Conflict of interest statement

The authors declare that the research was conducted in the absence of any commercial or financial relationships that could be construed as a potential conflict of interest.
